# Characterization and Ex Vivo Application of Indocyanine Green Chitosan Patches in Dura Mater Laser Bonding

**DOI:** 10.3390/polym13132130

**Published:** 2021-06-29

**Authors:** Francesca Rossi, Giada Magni, Roberto Colasanti, Martina Banchelli, Maurizio Iacoangeli, Erika Carrassi, Denis Aiudi, Alessandro Di Rienzo, Luca Giannoni, Laura Pieri, Stefano Dallari, Roberto Pini, Paolo Matteini

**Affiliations:** 1 Istituto di Fisica Applicata “Nello Carrara”, Consiglio Nazionale delle Ricerche, Sesto Fiorentino, 50019 Florence, Italy; f.rossi@ifac.cnr.it (F.R.); r.pini@ifac.cnr.it (R.P.); p.matteini@ifac.cnr.it (P.M.); 2 Department of Neurosurgery, Università Politecnica delle Marche, 60121 Ancona, Italy; neurotra@gmail.com (M.I.); erika.carrassi89@gmail.com (E.C.); denis.aiudi@gmail.com (D.A.); alessandrodirienzo1@gmail.com (A.D.R.); 3 Department of Neurosurgery, Padua University Hospital, 35128 Padua, Italy; 4 El.En. S.p.A., Calenzano, 50041 Florence, Italy; l.giannoni@elen.it (L.G.); l.pieri@deka.it (L.P.); 5 ENT Department, Ospedale “A. Murri”, 63900 Fermo, Italy; dallarinew@libero.it

**Keywords:** chitosan ICG-loaded patches, indocyanine green, laser bonding, dura mater

## Abstract

Dura mater repair represents a final and crucial step in neurosurgery: an inadequate dural reconstruction determines dreadful consequences that significantly increase morbidity and mortality rates. Different dural substitutes have been used with suboptimal results. To overcome this issue, in previous studies, we proposed a laser-based approach to the bonding of porcine dura mater, evidencing the feasibility of the laser-assisted procedure. In this work, we present the optimization of this approach in ex vivo experiments performed on porcine dura mater. An 810-nm continuous-wave AlGaAs (Aluminium Gallium Arsenide) diode laser was used for welding Indocyanine Green-loaded patches (ICG patches) to the dura. The ICG-loaded patches were fabricated using chitosan, a resistant, pliable and stable in the physiological environment biopolymer; moreover, their absorption peak was very close to the laser emission wavelength. Histology, thermal imaging and leak pressure tests were used to evaluate the bonding effect. We demonstrated that the application of 3 watts (W), pulsed mode (T_on_ 30 ms, T_off_ 3.5 ms) laser light induces optimal welding of the ICG patch to the dura mater, ensuring an average fluid leakage pressure of 216 ± 105 mmHg, falling within the range of physiological parameters. This study demonstrated that the thermal effect is limited and spatially confined and that the laser bonding procedure can be used to close the dura mater. Our results showed the effectiveness of this approach and encourage further experiments in in vivo models.

## 1. Introduction

Laser treatments are successfully used in different medical fields, such as in dermatology or ocular surgery [1,2,3,4,5,6]. In all these fields, laser systems have brought significant improvements and advantages for both the physicians and the patients, such as increased cooperation and reduction of pain and discomfort.

A widely exploited procedure combined with the use of an energy-based device is laser bonding. Nowadays, laser bonding is proposed as a valid alternative or, in addition to the classical suture for different tissues [7,8,9], the irradiation with selected wavelengths and the use of exogenous absorbers enabling a selective effect. In this respect, it has been widely demonstrated that laser-assisted closuring of wounds helps to reduce several issues related to the use of stitches and needles. Indeed, there is no foreign body reaction and needle trauma; the procedure results in a better and faster healing phase of a surgical wound, with an optimal reorganization of the treated tissue architecture. Furthermore, no scar formation and immediate watertight closure of the wounded tissue are observed, thus reducing the risk of infections [4,10,11,12].

The use of natural biopolymers for tissue repair has gained importance in the last few decades due to their widespread use in the biomedical and pharmaceutical fields. Natural polymers are more conducive to cell adhesion than synthetic ones and address a wide variety of biological needs: they can act as a support for in vitro cultured cells; a biocompatible matrix can be populated by different cell types and originate neo-tissues once implanted in the body [13,14].

Nevertheless, both natural biopolymers (e.g., collagen, elastin and fibrin) and polysaccharides or synthetic biopolymers, alone or in combination, have specific mechanical, physical and biological characteristics that make them crucial for this application [14]. In particular, polysaccharides as hyaluronan and chitosan and their derivatives are suitable for laser bonding, mainly for their high affinity to the biological matrix, biodegradability, non-toxicity and low cost. Moreover, chitosan is characterized by hemostatic, wound healing-promoting activity and naturally tends to form films with high mechanical strength and good elasticity [15,16,17,18,19,20].

The inclusion of indocyanine green (ICG), a dye frequently used as an absorbing chromophore for laser tissue welding, into the as-developed chitosan matrices has been exploited to construct tailor-made patches to enable the healing process and wound closure.

In previous studies, we published a preliminary ex vivo test of vocal fold laser bonding through ICG-infused chitosan patches, evidencing the feasibility of the laser-assisted procedure [21]. Subsequently, we proposed a new laser-based approach to bonding biological tissues: different biopolymeric matrices were designed and prepared in the form of a patch covering the surgical wound [12,22,23].

Recently, our attention has been drawn towards a challenge still open in the neurosurgical field: preventing postoperative cerebrospinal fluid (CSF) leaks following inadequate dural closures as this may produce significant complications (such as meningitis, neural tissue herniation, hypertensive pneumocephalus, pseudomeningocele, etc.) that substantially raise morbidity and mortality rates while increasing healthcare costs [24]. The dura mater is the outermost layer of the meninges that cover the central nervous system. It consists predominantly of collagen and elastin and possesses viscoelastic properties necessary to solve its biological functions [25,26,27]. Many biomaterials and sealants are used for dural closure, but the results are suboptimal. Since the dura mater is both a perfect impact absorber and a formidable barrier, the strength of the material, thickness, and elasticity represent crucial parameters for an ideal dural substitute [28]. In addition, an optimal substitute must be non-immunogenic, cheap, and should reduce infection occurrence [28].

In this scenario, we propose the use of chitosan in laser bonding application in dura mater as a valuable aid to overcome this challenge. We thus present the optimization of both the ICG-chitosan patches and the laser bonding approach for dura mater reconstruction in ex vivo experiments conducted in the porcine tissue.

## 2. Materials and Methods

### 2.1. Preparation of ICG-Loaded Solders

Indocyanine green (ICG) was purchased from PULSION Medical System (Munich, Germany, marketing authorization 700032978) and was used as received. Ethanol (product code 5199976), Chitosan low molecular weight (product code 448869-50G), and Hyaluronic Acid were purchased from Sigma-Aldrich (Milano, Italy). Physiological saline solution (0.9% NaCl) was from Eurospital (Trieste, Italy, marketing authorization 032182038). Milli-Q Ultrapure Water (Merck-Millipore Life Science, Milano, Italy) was used to prepare all aqueous solutions. Absorption spectra of the ICG-loaded solders solutions were obtained at room temperature in the 350–900 nm spectral range using a UV–Vis– NIR spectrophotometer (JASCO V-6, Jasco Europe, Lecco, Italy) with quarts cuvettes (QS, Hellma, Fisher Scientific, Milano, Italy, product code 10514782) of 1 cm and 1 mm optical path. The ICG-loaded chitosan patches were prepared by pouring a 30 μL chitosan 3.3% *w*/*v* dispersion in an aqueous acidic solution containing ICG (0.02% *w*/*v*) on circular polystyrene molds. After evaporation of the solvent, the samples were neutralized with 1 M NaOH solution (Sigma-Aldrich, Milano, Italy, product code S8045) and finally washed out with abundant water. The as-obtained patches (0.8 cm diameter, about 40-μm thickness) were carefully detached from the moulds and stored until their use in the experiments [21]. The UV–Vis absorption spectra of the ICG-loaded patches were measured at 25 °C and 4 °C temperatures and different times after their preparation to evaluate the stability and the optical properties of the material over time [21].

### 2.2. The Ex Vivo Porcine Model

The experiments were performed in the PhotoBioLab facilities (Firenze, Italy). Four porcine heads from nine-month-old male animals were harvested and purchased in the local slaughterhouse (Italpork, Pistoia, Italy, EC authorization n° IT702M). The heads were delivered to the lab within three hours post-mortem. A wide craniotomy of the skull was conducted by an experienced neurosurgeon, with the purpose of exposing the dura mater and the underlying cerebral cortex. After dura mater incision, a laser bonding duroplasty was performed in the treated group (*n* = 2), while conventional suturing was conducted in the control group (*n* = 2). In this last group, after the incision of the dura mater, its margins were closed using 3–0 silk sutures evenly spaced 3 mm apart and 3 mm from the incision edge itself.

### 2.3. Laser Bonding

The laser used for bonding is an AlGaAs (Aluminium Gallium Arsenide) diode laser (produced by Quanta System Spa, Milano, Italy), emitting at 810 ± 10 nm. The device is enclosed in a compact cabinet and equipped with a fiber optic, with different available inner core diameters ranging between 300 and 800 μm. The laser bonding procedure was performed with the aid of a surgical microscope. The dura mater was incised, a chitosan patch was put onto the external dura surface, then bonded with single laser spots. The weld line was approximately 1.5 cm long. The laser was equipped with a 550 μm diameter optical fiber, and a laser pulse burst was delivered to the patch to induce local patch/tissue adhesion. The fiber tip was kept in contact with the chitosan patch onto the tissue; the surface has been maintained hydrated during the treatment. The fluences applied were 10 J/cm^2^, 3 Watt (W) applied in continuous pulsed mode (T_on_ 30 ms, T_off_ 3.5 ms); 4 W, 250 ms and 4 W, 500 ms. Temperature control was performed using a thermal camera (Infrared Camera, R300SR, Nippon Avionics CO, LTD, Japan) during the laser bonding procedure: even if it is impossible to follow the temperature dynamics during treatment (as the fiber tip is in contact with the patch), it is possible to estimate the final temperature at the treatment end.

### 2.4. Fluid Leakage Pressure Test

Tests were performed to evaluate the leak pressure of the laser-bonded duroplasty. The specimens were tightly fixed to the rim of a 5-mL syringe (RAYS s.p.a., Ancona, Italy, product code 55LC) using a 2–0 silk suture (Ethicon Inc., Johnson & Johnson, Somerville, NJ, USA, product code K833H). The 5-mL syringe was filled with saline solution and was placed in series with a pressure transducer (usually employed for intracranial pressure monitoring, Integra LifeSciences Corporation, Plainfield, NJ, USA) and a 10-mL syringe that was used to gradually raise the water pressure until the fluid was noted to leak from the duroplasty. The fluid leakage pressure was read from the monitor connected with the pressure transducer.

### 2.5. Histology Examination

Samples were harvested after laser treatment and prepared for standard histology analysis. Where not otherwise specified, all reagents and consumables have been purchased from Bio-Optica, Milan, Italy. The specimens were immediately fixed in 10% neutral formalin (product code 05-01004F), dehydrated in the descending series of alcohol, cleared in Histo-Clear (National Diagnostics, Atlanta, GA, USA, product code HS-200), and embedded in paraffin (product code 08-7910). The hydration was performed in gradated series of alcohol: xylene (product code 06-1304F), absolute alcohol (product code 06-10099), 95% alcohol (product code 06-10090E), and 70% alcohol (product code 06-10075Q), followed by immersion in water for 5 min for 3–5 min each.

After these steps, the sample was immersed in Carrazzi’s hematoxylin (product code 05-M06012) for 15 min and an aqueous solution of 1% of eosin (product code 05-M10002) for 5 min; between the two-colour steps and at the end of the coloration, the samples are washed in tap water for 5 min. The dehydration was obtained using alcohol 70% (1 min), alcohol 95% (3 min), absolute alcohol (5 min), xylene (5 min). Finally, a clearing agent and Eukitt mount (product code 09-00100) were used. Microtome (Thermo Scientific Shandon Finesse ME+, Milano, Italy) equipped with R35 stainless microtome blades (Feather, pfm medical ag, Cologne, Germany, product code 207500005) were used to obtain several slices (4–5 μm thickness). Each slice was collected on microscope slides (Superfrost Plus, Epredia, Thermo Scientific, Milano, Italy, product code 10149870) for light microscopy evaluation. The blinded photographs were acquired by experienced personnel using a Nikon microscope (Nikon, Minato, Tokyo, Japan) through a digital photo camera (Nikon Digital Sight DS-U1) connected with a personal computer hosting the software Nis Elements D 3.2 (Nikon, Minato, Tokyo, Japan).

### 2.6. Statistical Analysis

For each experimental condition, photographs from five regularly spaced slices from the same sample were analyzed with the open-source software, ImageJ (U.S. National Institutes of Health, Bethesda, MD, USA, https://imagej.nih.gov/ij/, accessed on 5 February 2021). Quantitative data were shown as mean ± standard deviation (SD). The statistical analysis was carried out using GraphPad v.7 (GraphPad Software Inc., La Jolla, CA, USA). The data were obtained using one-way ANOVA analysis (Kruskal–Wallis test) followed Dunn’s post-hoc tests. The values were considered statistically significant when the *p*-values were less than 0.05.

## 3. Results

### 3.1. Optimization of ICG Optical Properties in Solder Solution

**Figure 1:**
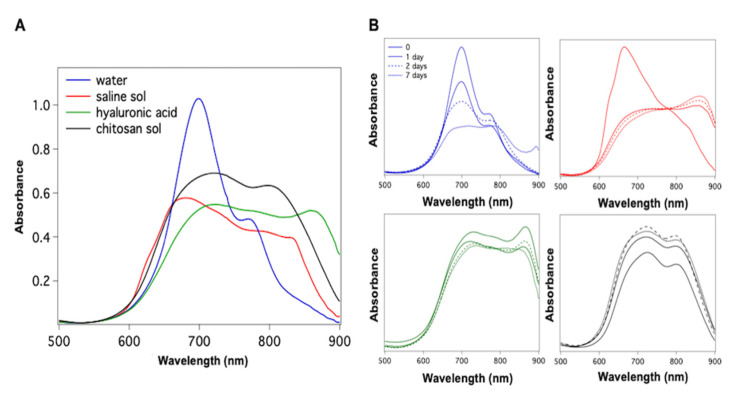
Absorption spectra of 0.02% ICG in various physiological media (**A**). Absorption spectra of (**A**) at the different aging time and stored at 25 °C (**B**).

The absorption properties of ICG were measured at different concentrations and in various solders solutions to find the absorption peak matching better the diode laser wavelength used for the bonding. In Figure 1A, the absorption spectra of 0.02% ICG dispersed in water, saline solution (0.9% NaCl), hyaluronic acid (3.3%), and chitosan solution (3.3%) are reported. Figure 1B shows the changes in the absorption spectrum over one week at 25 °C. In water, ICG has an absorption maximum at 695 nm with a shoulder at 780 nm, which corresponds to the absorbance of the aggregated and monomer form of ICG molecule, respectively [29]. For pure water, we observe poor stability of the dye; indeed, after 24 h, the absorbance of ICG decreases by about 25%. After 48 h, the absorbance of the solution falls steadily at 695 nm, and after one week, it is less than 50% of the original value. At 800 nm, there is a slight tendency for the absorbance to increase. After one week, also the absorbance at 900 nm, which is zero for a freshly prepared ICG solution, increases considerably. This new absorption maximum is ascribed to new ICG aggregates. The absorption spectrum of ICG in saline solution shows a maximum at 690 nm and shoulders at 780 nm and above 800 nm. At 25 °C, the system is not stable; in fact, at 24 h, an absorption maximum near 900 nm rapidly appears due to the formation of large ICG aggregates. The absorption spectrum of ICG in hyaluronic acid has two maxima of similar intensity at 724 nm and 780 nm, and a more intense band at 864 nm. The positions of the absorption maxima of ICG are shifted to longer wavelengths compared to their positions in the spectra in water and saline solution. The maximum at 864 nm originates from the aggregated species of the dye in the hyaluronic medium. The temperature of storage and aging time of the sample are weakly influencing ICG spectral properties: only a small decrease in the absorbance at 864 nm occurs at 25 °C after 48 h from the preparation of the sample. Therefore, the stability of the ICG dye in hyaluronic acid is greater than in an aqueous or saline solution.

In the absorption spectrum of ICG that was dispersed in the chitosan solution, two absorption maxima are present at 720 nm and 805 nm. In contrast, any other absorption band appears at longer wavelengths. The positions of the maxima are referred to as the absorption of ICG aggregates and monomer, respectively, which are red-shifted if compared to the positions in the aqueous system. Since no absorbance occurs at around 900 nm, no aggregates are revealed. The chitosan solution prevents second-order aggregation phenomena of the ICG dye. Our analysis indicates that the absorption peak of ICG associated with its monomeric form increases in the presence of chitosan, as it was observed previously, in the presence of serum proteins [30].

### 3.2. Characterization of ICG-Loaded Chitosan Patches

**Figure 2:**
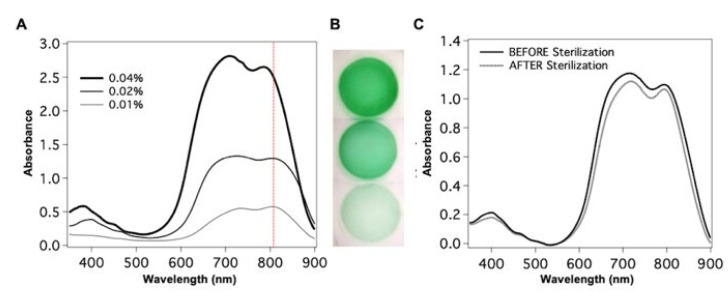
(**A**) Absorption spectra of ICG-loaded chitosan patches for 0.01%, 0.02% and 0.04% ICG; the red dashed line indicates the 810 nm wavelength of the laser used for the bonding experiments; (**B**) Images of freshly prepared ICG patches; (**C**) Absorption spectra of the ICG-loaded patches before and after the sterilization.

When 0.02% of ICG is incorporated in the 3.3% chitosan solid patch (thickness 40 μm), the absorption spectrum presents two bands centered at 720 nm and 808 nm as the chitosan solution. In this condition, the stability of the ICG optical properties results higher than in the chitosan solution; in fact, the absorption spectrum of ICG in the chitosan patch is unchanged over one week at 25 °C. The protection provided by the chitosan membrane is sufficient to prevent degradation of ICG, as it happens in water and saline solution. The absorption properties of the ICG-loaded chitosan patches were evaluated at different ICG concentrations, 0.01%, 0.02%, 0.04% (Figure 2A): the ICG concentration providing an absorption peak that matches better the wavelength of the laser welding is 0.02%. Patches were finally treated in a 70:30 ethanol–water mixture for 1 h for sterilization. We did not observe any change in the UV–Vis spectra before and after the treatment, as shown in Figure 2C.

Images of the as-prepared patches taken with a Dino-Eye Edge Digital Eye-Piece Camera (AM7025X, OK Italy S.R.L., Milano, Italy) are shown in Figure 2B. The patch colour appears homogeneous all over the area, and we noticed that the typical coffee-ring effect, commonly observed upon evaporation of the solvent from a drop of a liquid drying on a solid surface, is not observed here. This is likely due to the presence of the chitosan polymer in the evaporating solution.

### 3.3. The Ex Vivo Tests in Dura Mater Laser Bonding

**Figure 3:**
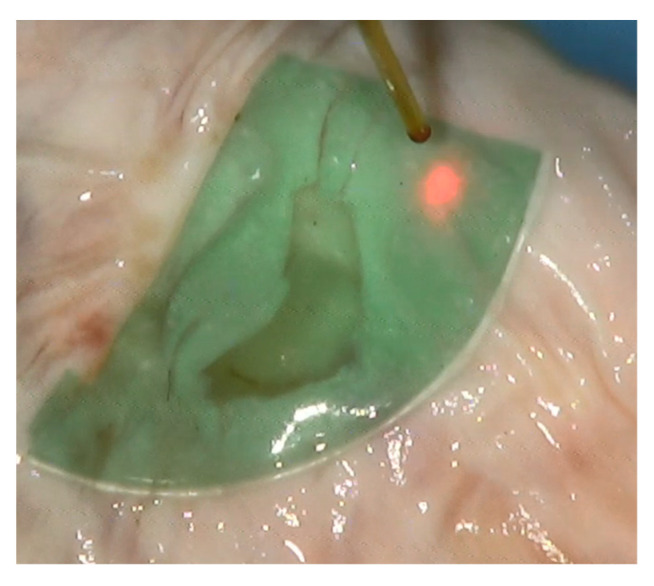
The laser bonding procedure. The green area is the ICG-stained chitosan patch. The red spot is the laser pointer; it is also visible the fiber optic used to deliver the 810 nm laser light. The white tissue is the dura mater positioned below the porcine brain. The patch is closing a hole in the dura mater to perform subsequent fluid leakage pressure test experiments.

**Figure 4:**
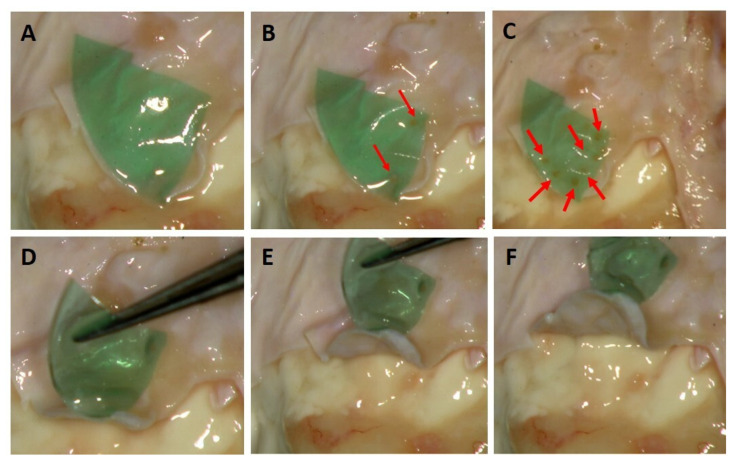
(**A**–**C**) Laser bonding of the dura mater. The red arrows indicate a characteristic green to beige transition of the patches, which proves the successful patch/tissue adhesion; (**D**–**F**) After the laser bonding, the ICG-loaded chitosan patch together with the welded porcine dura mater could be effectively raised using surgical forceps.

**Figure 5:**
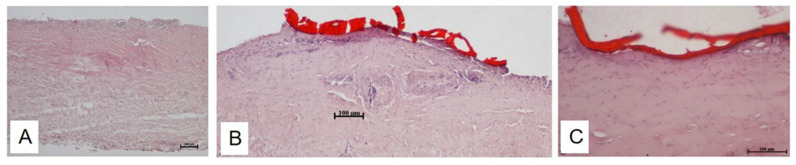
(**A**–**C**): Histological images of porcine dura mater. (**A**): Control, dura mater isolated and included to perform histological staining, without any treatment. (**B**,**C**): ICG patch (in red) welded on the surface of the dura mater, after the application of 3 W in pulsed mode, T_on_ 30 ms, T_off_ 3.5 ms. (**A**,**B**): 10×, (**C**):20×. Scale bar: 100 μm.

**Figure 6:**
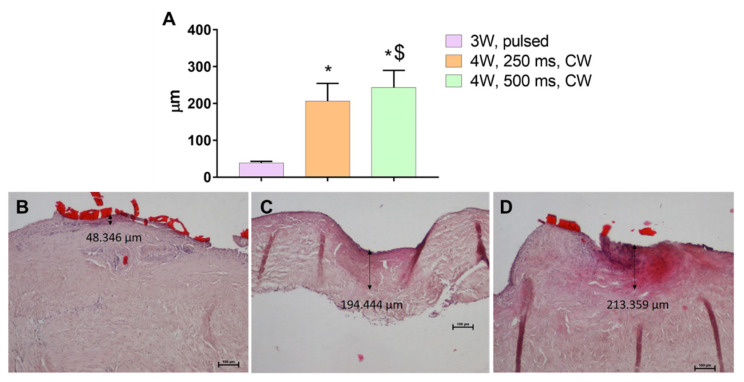
(**A**): Average of the thermal effect depth in the effective bonding of an ICG patch to the dura mater (violet column), with the use of a 3 W diode laser in pulsed mode (T_on_ 30 ms, T_off_ 3.5 ms); thermal damage depth in the tissue following the application of a 250 ms—4 W diode laser (orange column) and 500 ms—4 W (green column) in continuous wave (CW). Data are expressed as mean ± SD. Analysis was performed using one-way ANOVA, Kruskal-Wallis test followed by Dunn’s post-hoc multiple comparisons. Significant values: * *p* < 0.0001 vs. 3 W, pulsed; ^$^ *p* < 0.05 vs. 4 W, 250 ms. (**B**–**D**): representative histology of laser bonded porcine dura mater with the thermal damage depth measurement. The tissue was stained with hematoxylin and eosin. The bright red area is the ICG chitosan patch. Black arrows depicted the thickness of the optimal thermal effect (**B**) and thermal damage (**C**,**D**), represented by dark pink tissue. (**B**–**D**): 10X; Scale bar: 100 μm.

The laser bonding procedure (Figure 3) of the porcine dura mater using ICG-loaded chitosan demonstrates an immediate laser-induced adhesion of the ICG-infused chitosan patch. The effective laser bonding (obtained applying 10 J/cm^2^, 3 Watt (W), pulsed mode, Ton 30 ms, Toff 3.5 ms) was evidenced by a slight modification of the appearance of the green patches in the bonding site (a characteristic green-to-beige transition) (Figure 4). The histology performed on treated samples evidenced a good adhesion between the patch and the dura mater tissue, with a spatially confined and limited thermal effect in the tissue (38.39 ± 4.53 μm). A normal cortical cellular morphology and architectural pattern were observed under the laser bonding site (Figure 5). The mean fluid leakage pressure was 216 ± 104.91 mmHg (range, 100–350 mmHg). In the additional five experiments (N = 2), where the standard suture was applied, the closure of the dura was performed with 3–0 silk sutures, and the fluid leakage pressure test showed an immediate fluid leakage between the stitches and along the suture line in all the cases (data not shown). Figure 6 shows the extent of the thermal damage in the tissue induced by the application of 4 W per 250 ms or 500 ms, in continuous wave (Figure 6A) compared with the thermal effect that has instead guaranteed optimal welding of the ICG-patch and no damage to the underlying tissue (Figure 6B). In Figure 6C, the irradiation was delivered using a 535 μm diameter optical fiber; in this case, 4 W per 250 ms were used in single pulsed mode. This application did not allow the bond of the ICG-patch to the underlying tissue, and thermal damage is clearly visible and spread in the tissue on average for 206.6 ± 47.8 μm. In Figure 6D, a laser emission power of 4 W was applied, but with a time duration of 500 ms and using a 550 μm optical fiber; in this case, the average extent of thermal damage measured in the tissue was 243.6 ± 46.1 μm. These settings allowed the partial welding of the ICG-patch in some points of the tissue but with collateral thermal damage. However, in both cases, the thermal damages are spatially confined in the laser spot area.

### 3.4. Temperature Rise Due to the Laser Bonding Procedure

The maximum measured temperature after irradiation was consistently below 55 °C in all the different performed experiments (data not shown). As it was not possible to monitor the temperature dynamics during treatment (as the fiber tip was in contact with the patch), we controlled the temperature throughout the laser bonding procedure in the whole surgical scenario, and we measured the final temperature in the bonding area soon after the fiber tip was lifted from the patch. These observations were also validated by the tissue analysis through histology, evidencing the eventual thermal damage and its degree (Figure 5 and Figure 6).

## 4. Discussion

This study considers some issues concerning the laser bonding of biocompatible materials to the dura mater using an AlGaAs diode laser and ICG as an absorbing chromophore to localize laser heating to the weld surface, the biomaterial/tissue interface where bonding occurs. In comparison with other ICG-loaded solders, the ICG-loaded chitosan system allowed a synergistic effect of thermal and optical processes for the optimal laser welding procedure. The ICG-loaded chitosan patches were fabricated and optimized in composition and mechanical properties; they were resistant, pliable and stable in the physiological environment. Moreover, their absorption peak was in the near-infrared spectral region centered very close to 810 nm, thus matching the diode laser wavelength.

From the UV–Vis spectra measurements, we found that when ICG is incorporated in the chitosan patch, there is a reduction in ICG aggregational events with respect to aqueous and saline solder solutions. The presence of high levels of ICG monomers in the chitosan patches confers thermal stability to the system. A possible explanation is that upon binding to chitosan, ICG movement is hindered. The restricted molecular vibration and translation of ICG can minimize its conformational distortion and maintain its absorption properties. ICG-loaded patches thus preserve ICG optical properties from thermal stress. Similar results were achieved by encapsulating ICG into polymeric constructs or binding it to bovine serum albumin (BSA) [30].

On the other hand, recent studies reported that the monomeric absorbance of free ICG decreased by 55% over 48 h of incubation at 40 °C. In comparison, when ICG was encapsulated into constructs of poly-allylamine hydrochloride, there was only a 10% reduction in monomer absorbance under the same conditions [31]. Our findings with the ICG-chitosan patches reiterate the importance of the polymer in maintaining the monomeric absorbance at physiological temperature. The enhancement and the stability of ICG monomer absorbance in the ICG-chitosan patches have implications in phototherapeutic applications of this material, such as photothermal procedures or photodynamic therapy (PDT), as previously demonstrated [11,32,33].

In the present study, some considerations must be given to the appropriate choice of the radiant exposures: for instance, the increased absorbance may allow the use of lower radiant exposure and can be utilized in several biomedical fields, such as neurosurgery applications. An inadequate dural closure after a neurosurgical operation remains a significant concern, as it may determine detrimental clinical consequences, as well as an increase in healthcare costs. Dural substitutes and sealants allow lowering the overall incidence, but they are expensive and associated with various complications, both acute and chronic. Consequently, a standardization of dura mater reconstruction is still lacking [21].

Several fundamental properties, such as excellent biocompatibility and biodegradability, make chitosan a very suitable material for laser bonding [19,20]. Chitosan naturally tends to form films with high mechanical strength, good elasticity, and relatively slow biodegradation, promoting a better reorganization of the tissue in the postoperative period [19,20]. Besides, the inclusion of the chromophore (ICG) in the biopolymeric matrices to be bonded may help decrease the rise in the local tissue temperature and to improve the spatial selectivity of the procedure [34]. However, there have been few reports so far of chitosan used as a substitute for dura mater [32,35]. Sandoval-Sánchez et al. compared in a New Zealand rabbit model three different dural reconstruction techniques: primary suture closure of the autologous dura mater, duroplasty with a non-suturable collagen matrix duroplasty with a bilayer chitosan scaffolding fixed with sutures. Chitosan allowed effective dural repair without a cerebrospinal fluid leak. No significant differences in fluid leakage pressures between the three groups were reported. Moreover, chitosan promoted an organized regeneration with fibroblasts without evidence of fibrosis [32]. Tissue repair with laser-activated chitosan films has been proposed [36,37,38], but there have been no reports of their use for dural closure [21].

In a previous study, we demonstrated the feasibility of the laser bonding technique for dura mater reconstruction in an ex vivo porcine model using ICG-infused chitosan patches [21]. Here, we optimized the procedure in four tissue samples of dura mater: in two samples, the conventional suture was used, while the other two were used to test different irradiation parameters to identify the best ones and proceed with leakage pressure tests, histological analysis, and thermal imaging acquisition.

Regarding identifying the optimal irradiation parameters, we started by testing the laser varying the output power, the emission mode, and the irradiation time, based on our previous experiences [21], also performed in other biological tissues [6,11]. During the tests, we excluded all the irradiation parameters that induced clear carbonization of both the tissue and the ICG patch. Furthermore, we also eliminated those measures that did not stimulate an optimal dura mater—patches welding, e.g., where the spots did not exhibit the classic transition of the ICG color from green to beige and where the bonding points are not well placed on the underlying tissue. We identified three irradiation conditions from these tests that did not induce visible thermal damage and guaranteed a suitable sealing of the ICG patch to the dura mater.

The histological analysis performed on samples irradiated using 3 W, T_on_ 30 ms, T_off_ 3.5 ms applying a fluence of 10 J/cm^2^ evidenced a good adhesion between the chitosan patches and the dura mater in our experimental conditions. This fluence induces a negligible and spatially confined thermal effect (38.39 ± 4.53 μm). Moreover, the brain tissue underlying the laser bonding site had a regular pattern of neurons and glial cells that showed typical histological features. In the other two conditions tested—4 W for 250 ms and 4 W for 500 ms, using fiber of 535 and 550 nm in diameter, respectively—the adhesion of the patch was not uniform, and, above all, the histology showed pervasive thermal damage in the tissue (206.6 ± 47.8 μm and 243.6 ± 46.1 μm, respectively)

The fluid leakage test was performed only in samples where bonding between dura mater and ICG patch was excellent. In samples where this did not occur, this test failed, and no measurement was obtained. The mean fluid leakage pressure was 216 ± 105 mmHg, resulting in an immediate watertight dural closure without any standard suturing. Even if the range of the registered fluid leakage pressures was quite wide (100–350 mmHg), thus underlining the need for further standardization of the laser bonding procedure, the lowest value was potentially sufficient to withstand a sudden rise in intracranial pressure, i.e., during coughing or Valsalva’s maneuver. In other words, the laser-welded dural closure easily exceeded the normal range of the intraoperative cerebrospinal fluid pressure, whereas conventional suture closure did not.

The maximum measured temperature after irradiation (<55 °C) was, by far, lower than those reported in the previous studies on dural closure with laser tissue welding.

To summarize, our results obtained from ICG-loading patches characterization, histological analysis, thermography, and fluid leakage pressure test demonstrated that the laser-assisted technique for dural reconstruction provides an efficient dural closure in the ex vivo porcine model.

## 5. Conclusions

This study exploited a material known for its use in laser welding and laser bonding and an exogenous chromophore that showed a peak absorption in the wavelength of interest (810 nm). These ICG-loaded patches, thanks to their peculiar features, have been optimized to obtain the best bonding effect and a thermally stable system, with the absence of chitosan aggregates, usually found in aqueous formulations. The patches were tested in an ex vivo model to optimize a laser bonding technique as an excellent solution to the severe problem of dural fistulae. Histological analysis, thermography, and fluid leakage pressure tests showed that the ICG-loaded patches are functionally bonded to the dural tissue and can be proposed when the conventional suture technique is risky or complicated: when working through narrow and deep surgical corridors (e.g., key-hole or endoscopic approaches), in case of reoperation, or following radiotherapy. In addition, the use of chitosan eliminates problems related to sutures and the presence of foreign bodies that could induce infection and further complicate the postoperative period. These results encourage the use of this technique in the in vivo model to deepen knowledge and provide a usable alternative or support to a challenge still open in neurosurgery.

## Data Availability

The data presented in this study are available on request from the corresponding authors. The data are not publicly available, due to ongoing studies.
